# The Double‐Edged Sword of Perseverance: Active Coping, Grit, and Cognitive Health in Black American Men

**DOI:** 10.1002/alz70861_108614

**Published:** 2025-12-23

**Authors:** Darlingtina Esiaka, Candidus Nwakasi, Chizobam Nweke, Roland J. Thorpe, Elizabeth Luth

**Affiliations:** ^1^ University of Kentucky Sanders‐Brown Center on Aging, Lexington, KY USA; ^2^ University of Kentucky College of Medicine, Lexington, KY USA; ^3^ University of Connecticut, Storrs, CT USA; ^4^ Johns Hopkins Alzheimer’s Disease Resource Center for Minority Aging Research, Bloomberg School of Public Health, Baltimore, MD USA; ^5^ Robert Wood Johnson Medical School, Department of Family Medicine and Community Health, New Brunswick, NJ USA

## Abstract

**Background:**

Subjective cognitive decline (SCD) is increasingly recognized as a potential early indicator of dementia risk. Despite this, limited research has explored how psychological resilience factors, such as active coping and grit, relate to SCD—particularly among Black American males, a group often underrepresented in cognitive health research.

**Method:**

Black American males (*N* =138) completed validated questionnaires measuring SCD, active coping, grit, and demographic characteristics. Linear regression analyses were conducted to assess the independent and interactive effects of the predictor variables.

**Results:**

Both active coping (β = −0.287, *p* < 0.001) and grit (β = 0.630, *p* < 0.001) showed significant associations with SCD after adjusting for demographic factors (F[6,131] = 8.609, *p* < 0.001). Furthermore, grit significantly moderated the relationship between active coping and SCD (β = 0.153, SE = 0.02, 95% CI: [0.00, 0.08], *p* = 0.045).

**Conclusion:**

These findings highlight the importance of considering both risk and protective psychological factors in understanding cognitive decline among Black American males.

